# Supplementary material for: Precision Engineering of the Transcription Factor Cre1 in Hypocrea jecorina (Trichoderma reesei) for Efficient Cellulase Production in the Presence of Glucose
Source: Front Bioeng Biotechnol. 2020 Jul 28;8:852. doi: 10.3389/fbioe.2020.00852 (PMC7399057; doi:10.3389/fbioe.2020.00852)
Supplement: Supplementary file 1 [file Data_Sheet_1.docx]

Supplementary Material

# Supplementary Figures and Tables

## Supplementary Figures

**Figure legends**

**Supplementary Figure S1**. (a) A schematic diagram of the *cre1*^S387V^，*cre1*^S388V^，*cre1*^T389V^，and *cre1*^T390V^ deletion cassette replacement cassettes. X represents S387, S388, T389, and T390. The mutation of transformations was verified using PCR (b) and DNA sequencing (not shown).

## Supplementary Tables

**Table legends**

**Supplementary Table S1**. Primers used for strain construction in this study

**Supplementary Table S2**. Plasmids and cassettes used in this study


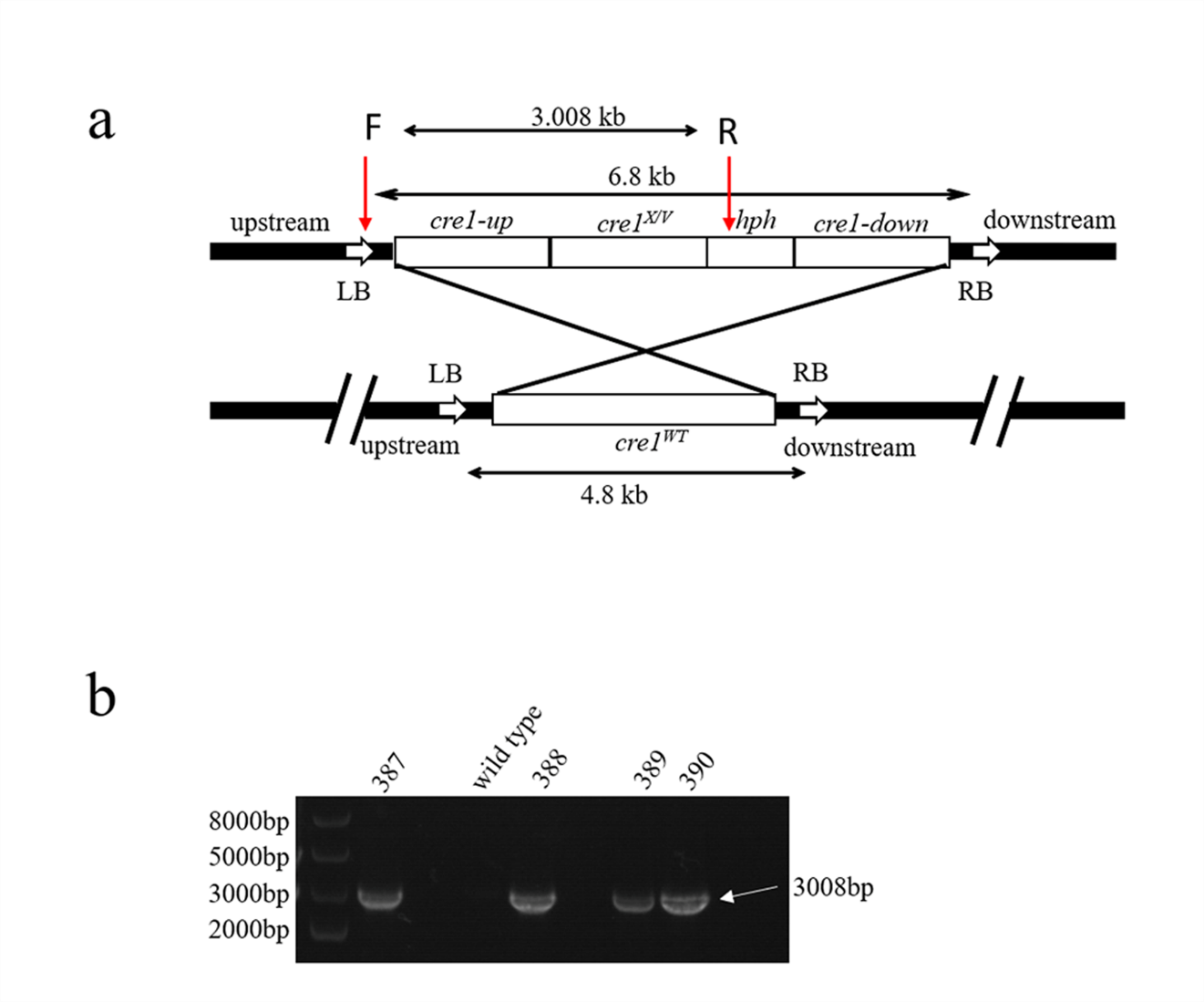


**Supplementary Figure S1**

**Supplementary Table S1**

| **Primer name** | **Sequence 5′–3′** |
| --- | --- |
| **P-Cre1-F** | AAGTTCCACCACGGGCAGTCTTGCCGGTGGCGACCTCATGGATC |
| **P-Cre1-R** | CAAGACTGCCCGTGGTGGAACTTCTGCCGCTGTTGGGGAAGA |
| **Cre1-F** | ATGCTACGAATCGAAGTACCGACC |
| **Cre1-F** | CACCCCCAAGCCAAGCTTCTAC |
| **Cre1-cx-F** | TCACACTCCCATCGCAACTCCC |
| **Cre1-cx-R** | ACATAACCTTCTGGCATGGCAGAC |
| **Cre-CS-F** | CAGAACCACACTCACTCACACACC |
| **Cre-CS-R** | AACACCGCGTCTTACGATATGTGA |
| **Cre-d-F** | TTGTACAAATAAGATCCACTTAACGTTACTGAAATCATCA |
| **Cre-d-R** | CACCCCCAAGCCAAGCTTCTAC |
| **Cre-up-F** | ATGCTACGAATCGAAGTACCGACC |
| **Cre-up-R** | CCTGCAGCCCGGCATCCGATCCATGAGGTCGCCA |
| **Cre0-F** | AGTTCCACCACGGGCAGTCTTGCCGGTGGCGACCTC |
| **Cre0-R** | ACTGCCCGTGGTGGAACTTCTGCCGCTGTTGGGGAAGA |
| **Cre387-F** | GTCTCCACCACGGGCAGTCTTGCCGGTGGCGACCTC |
| **Cre387-R** | ACTGCCCGTGGTGGAGACTCTGCCGCTGTTGGGGAAGA |
| **Cre388-F** | AGTGTCACCACGGGCAGTCTTGCCGGTGGCGACCTC |
| **Cre388-R** | ACTGCCCGTGGTGACACTTCTGCCGCTGTTGGGGAAGA |
| **Cre389-F** | AGTTCCGTCACGGGCAGTCTTGCCGGTGGCGACCTC |
| **Cre389-R** | ACTGCCCGTGACGGAACTTCTGCCGCTGTTGGGGAAGA |
| **Cre390-F** | AGTTCCACCGTCGGCAGTCTTGCCGGTGGCGACCTC |
| **Cre390-R** | ACTGCCGACGGTGGAACTTCTGCCGCTGTTGGGGAAGA |
| **RH-F** | GCCGACCGGATCAATGTCCGGTACTCATGGCGC |
| **RH-R** | AGTACCGGACATTGATCCGGTCGGCATCTACTCTATTC |
| **F** | ATCTCACTTGGTGAGCCCTGT |
| **R** | AATAATGTCCTCGTTCCTGTCTGCT |
| **actin-F** | TTAAGAAAGCCGCCACCCCC |
| **actin-R** | GTTGGTCGACAGGGAGAGGATG |
| **cbh1-F** | CTGCGACTGGAACCCATACC |
| **cbh1-R** | AAGTGACGCCATTCTGGACAT |
| **xyr1-F** | ACAGTGGAGCGGTAACAGACA |
| **xyr1-R** | CACGAATCCTTCCGACGAG |
| **clr1-F** | TCTTCCAACCCGTCTACGCA |
| **clr1-R** | ACCAGTACACGGCCTGTTCT |
| **clr2-F** | CGCCCAAGGATGATCAGCAG |
| **clr2-R** | GACTTTGACGGACGAGGCAC |

| Plasmids/cassettes | Features | Source |
| --- | --- | --- |
| pUG6-M | high-copy-number ColE1/pMB1/pBR322/pUC ori; *Amp* | Store in lab |
|  |  |  |
| pUG-Cre1 | pUG6-M carrying *P_Cre1_-Cre1-T_Trpc_-P_Trpc_-Hyg-T_Cre1_* cassette | This study |
|  |  |  |
| pUG-Cre1^S387V^ | pUG6-M carrying *P_Cre1_-Cre1*^S387V^*-T_Trpc_-P_Trpc_-Hyg-T_Cre1_* cassette | This study |
|  |  |  |
| pUG-Cre1^S388V^ | pUG6-M carrying *P_Cre1_-Cre1*^S388V^*-T_Trpc_-P_Trpc_-Hyg-T_Cre1_* cassette | This study |
|  |  |  |
| pUG-Cre1^T389V^ | pUG6-M carrying *P_Cre1_-Cre1*^T389V^*-T_Trpc_-P_Trpc_-Hyg-T_Cre1_* cassette | This study |
|  |  |  |
| pUG-Cre1^T390V^ | pUG6-M carrying *P_Cre1_-Cre1*^T390V^*-T_Trpc_-P_Trpc_-Hyg-T_Cre1_* cassette | This study |

**Supplementary Table S2**
